# Acute Thrombotic Occlusion Induced by an Inferior Vena Cava Filter Implanted 13 Years Earlier, Treated with the Indigo System and Filter Retrieval

**DOI:** 10.3400/avd.cr.26-00047

**Published:** 2026-08-29

**Authors:** Hitoshi Anzai, Keigo Kajiwara, Rikako Saito, Takehiro Kato, Hiroya Watanabe, Masaya Yuzawa, Mitsuyo Ito, Yusuke Samajima, Chihiro Fukuyama, Kento Yabe, Hiroki Takenaka, Naohiko Nemoto

**Affiliations:** Cardiology Department, SUBARU Health Insurance Ota Memorial Hospital, Ota, Gunma, Japan

**Keywords:** inferior vena cava filter, Indigo system, filter retrieval

## Abstract

A patient in his 40s, who had experienced acute deep vein thrombosis (DVT) of his left leg 13 years ago, developed acute DVT of his right leg due to an inferior vena cava filter (IVCF) thrombotic occlusion. A thrombus extended from just below the IVCF to the bilateral femoral veins. Given the limited availability of urokinase and the strict guidelines for the proper use of the Indigo CAT8 in Japan, we first performed catheter-directed thrombolysis with alteplase for 2 days, followed by aspiration with the CAT8 catheter after confirming the absence of an IVC thrombus. Finally, we successfully retrieved this IVCF, implanted 13 years earlier, using the bidirectional technique.

## Introduction

Inferior vena cava (IVC) filter (IVCF) placement has been used to reduce the risk of pulmonary embolism in high-risk patients. However, IVCF placement also entails risks, including migration, IVC wall penetration, fracture, and IVCF thrombosis.^1)^ IVCF thrombosis is a common complication after IVCF placement, reported to occur anywhere between 1.3% and 31%.^2,3)^ IVCF thrombosis is often asymptomatic but can present with lumbar pain, increased edema of the lower extremity, nonhealing venous ulcers, or renal dysfunction. Once it occurs, endovascular treatment for filter-associated ilio-caval thrombosis can be challenging, particularly when filter retrieval is difficult due to the management of the indwelling filter and the restoration of adequate flow through the IVC.^4)^

In this report, we presented a 40-year-old male patient with acute thrombotic occlusion of an IVCF (OptEase; Cordis, Miami Lakes, FL, USA) implanted 13 years earlier, which was successfully treated with restoration of blood flow via catheter-directed thrombolysis (CDT) with alteplase, followed by thrombus aspiration using an Indigo aspiration system CAT8 catheter (Penumbra, Alameda, CA, USA). Finally, the IVCF was retrieved using an advanced technique.

## Case Report

In 2012, the patient in his 20s experienced acute deep vein thrombosis (DVT) of the left leg and underwent IVCF placement (OptEase; Cordis). Since then, he has been taking anticoagulants regularly. He started taking warfarin first and then changed to a direct oral anticoagulant (Rivaroxaban 15 mg per day) in 2016. In May 2025, he experienced sudden severe pain and swelling in his entire right leg, which was on the opposite side of the previous DVT. He visited another hospital, and a computed tomography (CT) scan identified massive thrombus formation just below the IVCF with bilateral iliac vein thrombotic occlusion. Also, the CT clearly demonstrated compression of the left common iliac vein (CIV), a condition known as May–Thurner syndrome. They confirmed that he had been taking a direct oral anticoagulant constantly. He was admitted to that hospital, and an intravenous heparin infusion was started instead of a direct oral anticoagulant. The APTT was maintained between 42 and 68 seconds during this hospitalization. Five days later, he was transferred to our hospital, hoping for further treatment because he still had disabling right leg symptoms.

At the time of admission, his body weight was 60.2 kg, and his renal function was normal (Cr 0.61 mg/dl). The APTT was 48.0 seconds, and the D-dimer level was 8.6 ug/ml. As for the potential thrombotic predispositions, antiphospholipid antibody syndrome, antithrombin III deficiency, and protein S or C deficiency were evaluated at the previous hospital and at our hospital, and they were all negative.

**Fig. 1:**
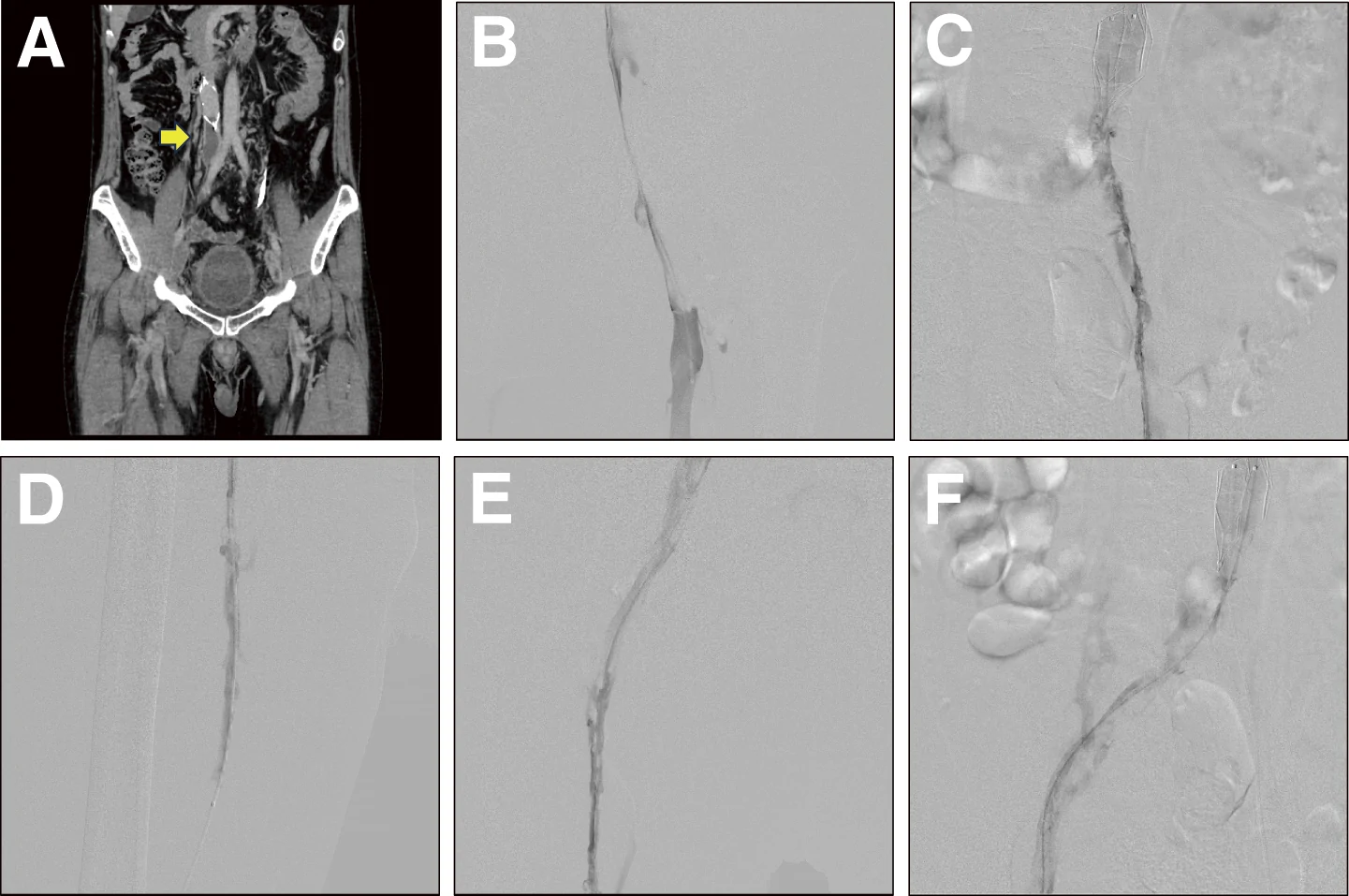
A CT shows thrombus formation in the IVC just below the IVCF (**A**). A venography through a Fountain catheter. Each venography shows a large amount of thrombus in the right proximal femoral vein to the right iliac vein (**B**, **C**), and in the left distal femoral vein to the left iliac vein (**D**–**F**). The IVC below the IVCF contains a large amount of thrombus. Because of the prone position, (**B**) and (**C**) show the right leg vein, and (**D**–**F**) show the left leg vein. IVC, inferior vena cava; IVCF, inferior vena cava filter

The severity of DVT was assessed: the r-VCSS pain score was 3, and the Villalta score was 15 in the right leg, while he did not complain of any symptoms in the left leg. Since the thrombus was in the IVC below the IVCF (**Fig. 1A**), we were unable to use the Indigo system because it violated the current appropriate-use guideline determined by the 4 Japanese academic societies (Japanese Society of Interventional Radiology, the Japanese Association of Cardiovascular Intervention and Therapeutics, Japanese Society for Vascular Surgery, Japanese Society of Phlebology). Therefore, we first performed CDT using alteplase, evaluated the presence or absence of residual thrombus, and then considered whether to perform additional thrombus aspiration therapy with the Indigo system. We obtained approval for the off-label use of alteplase as CDT from our hospital’s institutional review board (approval number: OR 22020) on June 1st 2022, just after Urokinase suddenly became unavailable in our country.^5)^ Regarding the procedure of CDT with alteplase, we administered alteplase continuously at 0.01 mg/kg/hour via an infusion catheter with multiple side holes (Fountain catheter: Merit Medical, South Jordan, UT, USA). This administration rate was determined based on the previous 2 major international randomized trials (CAVENT, ATTRACT).^6,7)^ CDT with alteplase was started after the patient was sent to the High Care Unit. During administration, we carefully monitored the patient by checking fibrinogen, hemoglobin, and APTT levels 3 times a day. We obtained written consent before the procedure for the off-label use of alteplase.

The first procedure (**Fig. 1**): we introduced a 4-Fr sheath into each popliteal vein under ultrasound guidance in a prone position. Then, a Fountain catheter (Merit Medical), which was a special multi-hole infusion catheter for CDT, was inserted into each 4-Fr sheath. We started administering alteplase at 0.2 mg/hr from the left side and 0.4 mg/hr from the right side (total amount was 0.6 mg/hr) through Fountain catheters and continued CDT for 2 d.

**Fig. 2:**
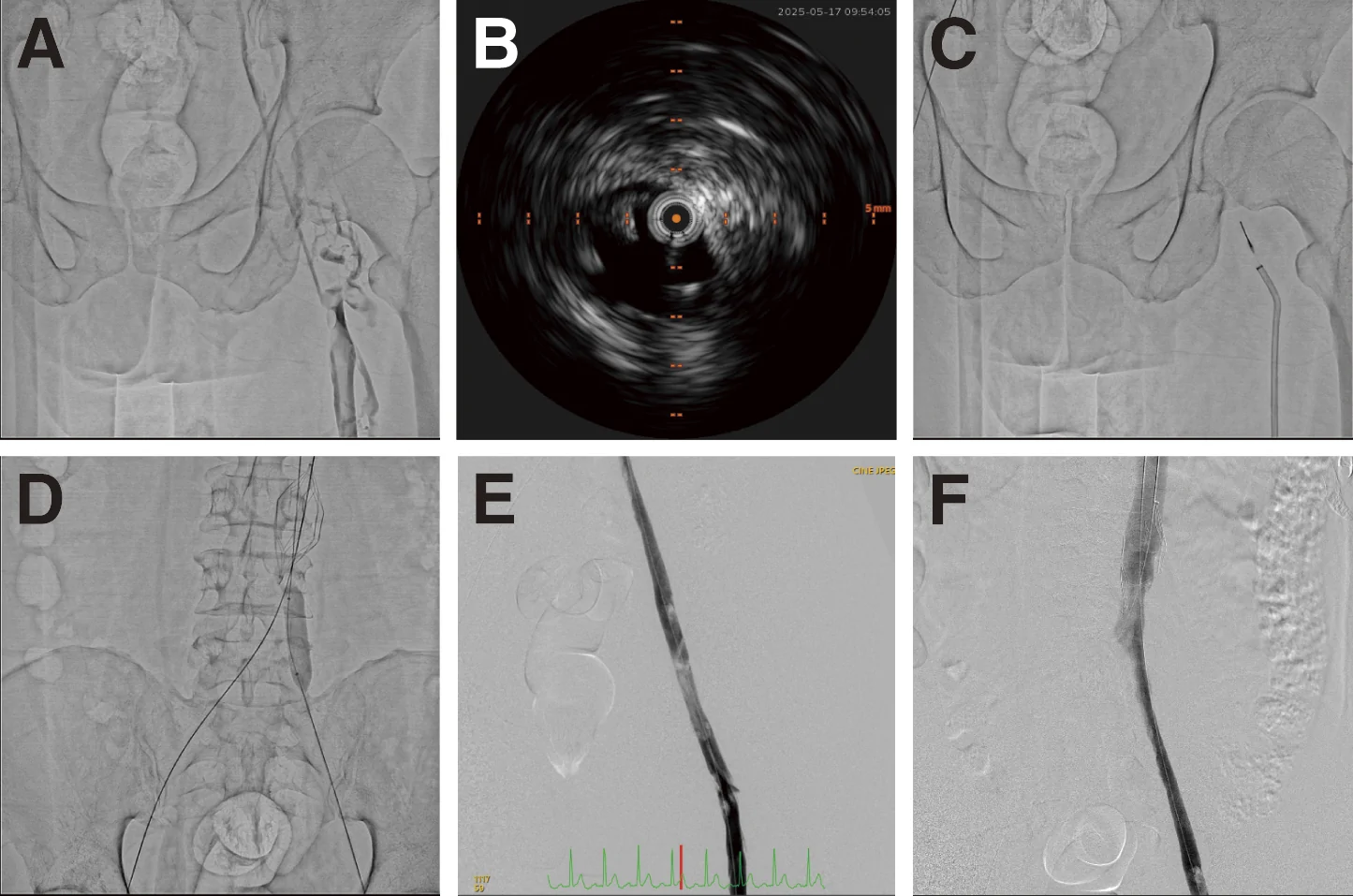
A venography shows a large amount of residual thrombus in the right leg vein, though slight antegrade flow is restored (**A**). IVUS reveals thrombus below the IVCF in the IVC disappeared (**B**). The residual thrombus in the right leg is aspirated with a CAT8 catheter (**C**). The right iliac vein and common femoral vein are dilated with a balloon with a 12-mm diameter (**D**). The final venography shows restoration of antegrade blood flow, with a little residual thrombus (**E**, **F**). Because of the prone position, each venography shows the right leg vein. IVUS, intravascular ultrasound; IVC, inferior vena cava; IVCF, inferior vena cava filter

The second procedure (**Fig. 2**): we removed the Fountain catheters and exchanged the 4-Fr sheaths for 9-Fr sheaths. A venography showed a reduction in thrombus volume, but there was still a residual amount of thrombus on both sides. We decided not to put a stent in the left iliac compression site and to leave the thrombus on the left side since the patient did not have any symptoms in the left limb. As for the right side, intravascular ultrasound (IVUS) showed a disappearance of the thrombus below the IVCF in the IVC. Then, we introduced a CAT8 catheter and aspirated the residual thrombus, followed by dilatation with a 12-mm-diameter balloon in the right iliac and femoral veins. The final venography showed excellent antegrade blood flow with a small residual thrombus. We obtained hemostasis at the puncture sites of both popliteal veins by using Perclose Prostyles (Abbott, Abbott Park, IL, USA).

**Fig. 3:**
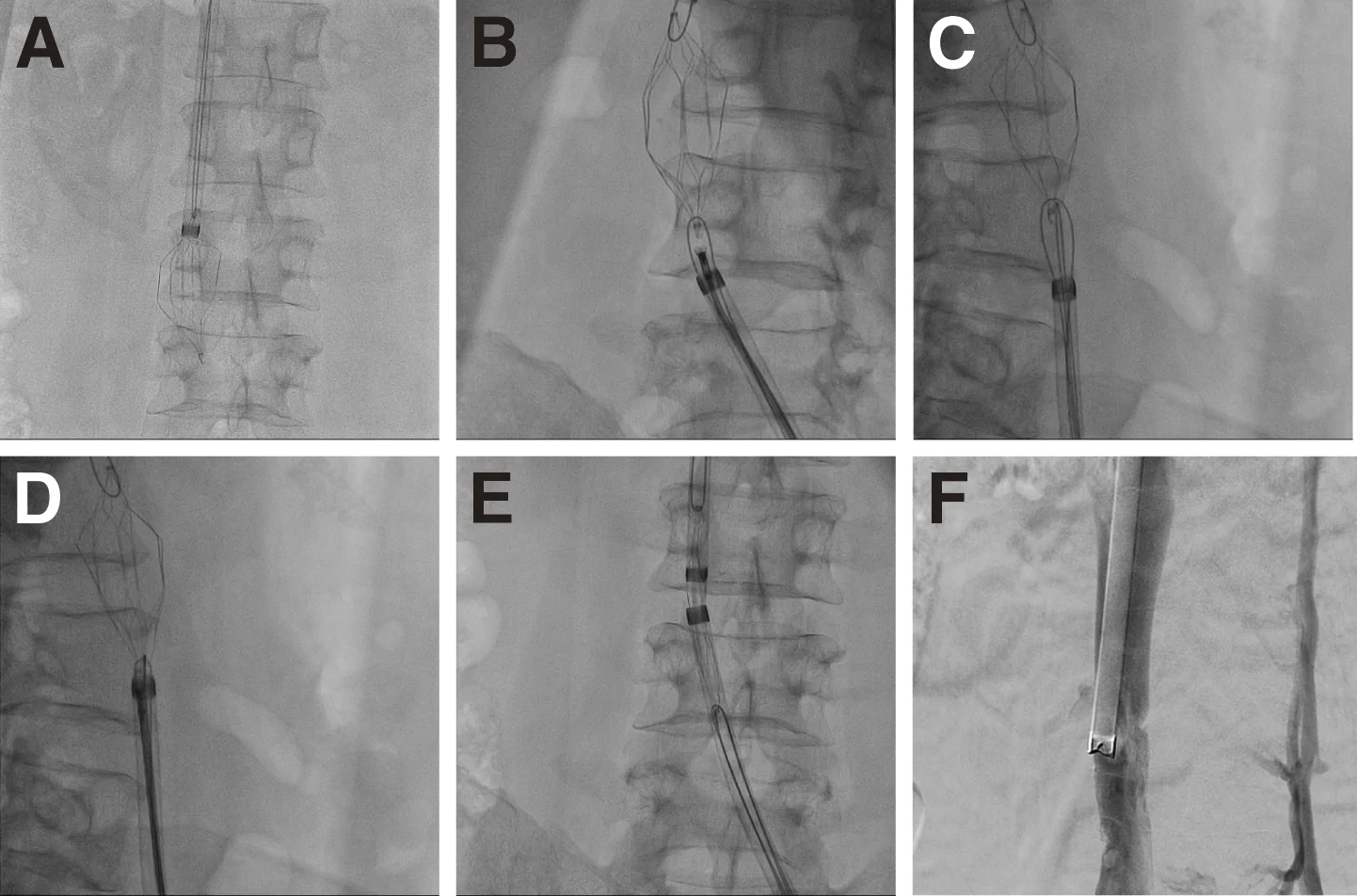
A sling is created with a 0.035 guidewire around the top of the IVCF, and the top of the IVCF is introduced into a 16-Fr guide sheath via the right internal jugular vein (**A**). The sling is created with a 0.035 guidewire around the bottom of the IVCF. Due to the embedded hook, the biopsy forceps cannot catch the hook (**B**). The second sling is created, and the bottom of the IVCF is successfully introduced into a 16-Fr guide sheath via the left common femoral vein (**C**, **D**). The 16-Fr guide sheath is advanced from either side. The ends of 2 guide sheaths touch together, and the IVCF is successfully withdrawn from the left common femoral vein (**E**). The final venography reveals no vessel injury (**F**). IVCF, inferior vena cava filter

The third procedure (**Fig. 3**): 4 days later, we attempted to retrieve the IVCF. Under general anesthesia, we applied a bilateral sling technique from the right internal jugular vein and the left femoral vein as well.^8,9)^ At each site, we advanced a 16-Fr, 70-cm Check-Flo guide sheath (Cook Medical, Bloomington, IN, USA). A sling was established with a 0.035 guidewire around the top of the IVCF; we created a loop around the top of the IVCF with a 0.035 guidewire supported by a 4-Fr JL1 diagnostic catheter. The 0.035 guidewire was caught by an EnSnare (Merit Medical), withdrawing the guidewire out of the sheath, and the sling technique was established. Then, the top of the IVCF was introduced into a 16-Fr guide sheath via the right internal jugular vein. Next, the sling was created with a 0.035 guidewire around the bottom of the IVCF. Due to the embedded hook, a 7-Fr biopsy forceps, ordinarily used for endomyocardial biopsy, was introduced; however, it could not grasp the embedded hook. The second loop was created, and the bottom of the IVCF was successfully introduced into a 16-Fr guide sheath via the left common femoral vein. The 16-Fr guide sheath was advanced from either side. The ends of 2 guide sheaths touched together, and the IVCF was successfully withdrawn from the left common femoral vein. The final venography did not reveal any vessel injury.

After these procedures, his leg symptoms disappeared dramatically. At 6 months after the procedure, a CT showed no thrombus in the IVC through the bilateral iliac veins. He has been asymptomatic and uneventful on Rivaroxaban 15 mg per day for 1 year after the procedure at the outpatient clinic in our hospital. Although the IVCF was removed, we planned to give him a direct oral anticoagulant indefinitely because he developed acute proximal DVT twice and had an unfixed compression of the left CIV (May–Thurner syndrome).

## Discussion

Although the IVCF insertion rate has gradually decreased due to fear of potential long-term complications, the retrieval rate remains low.^10)^ The IVCF thrombotic occlusion is one of the potential complications and can cause serious problems, including acute massive DVT, as in this case.

The current domestic issues in Japan regarding how to treat thrombotic peripheral vascular disease are the lack of an approved thrombolytic agent and a novel thrombus aspiration/removal device. As for the thrombolytic agent, UK, the only thrombolytic agent approved in Japan, suddenly became unavailable in 2021. Tissue-type plasminogen activator (t-PA) is approved only for cerebral stroke, acute pulmonary embolism, and acute myocardial infarction in Japan. After this shocking event, the Indigo aspiration system became covered by health insurance in Japan in September 2023.^5)^ However, the current appropriate-use guideline determined by the 4 Japanese academic societies states that it can be used when the thrombus does not extend into the IVC by more than 1 cm.

Given these reasons, we decided to perform CDT with alteplase first for 2 d in this case. At our institute, the local Institutional Review Board approved the use of t-PA for the treatment of thrombotic peripheral vascular disease as mentioned above. Then, after confirming the absence of the thrombus in the IVC below the IVCF, we used the CAT8 catheter to perform aspiration. We believe this strategy was successful amid the current confusing conditions in Japan. However, we could have skipped the CDT if the CAT8 catheter had been allowed for use in the event of an IVC thrombus.

Another critical issue in this case was whether the IVCF should be removed. The first point to discuss is that conservative management with anticoagulation alone would be an alternative, especially given the high risk of vessel injury when retrieving a filter embedded for 13 years. The reason we believed the OptEase needed to be retrieved was that there have been several reports that the box-shaped IVF, such as the OptEase, was more likely to develop thrombus formation in an IVCF.^11)^ Also, they said that filter strut fracture occurred more frequently compared to the conical-shaped IVCF.^12)^ Therefore, retrieval should be considered when clinically appropriate and technically feasible.

The second point is how to reduce the IVCF-associated complications, including recurrence of IVCF thrombotic occlusion like this case. There seemed to be 2 possible options: one was to retrieve the IVCF, and the other was to place a stent across the IVCF. Chick et al. demonstrated the result of endovascular treatments of thrombotic occlusion of the 123 IVCFs in 120 patients: acute in 58 patients and chronic in 62 patients.^13)^ The mean indwelling time of IVCF was 53 months, ranging from 3 d to 170 months. Thirty filters (24%) were retrieved, while 93 (76%) were excluded with stent placement across the indwelling IVCF. They concluded that technical and clinical outcomes in patients undergoing filter retrieval were similar to those in patients undergoing filter exclusion with a stent. One thing we needed to emphasize was that 53 (43%) of IVCFs were permanent-type, so they might have chosen not to retrieve the IVCF; they may have just placed a stent across the occluded IVCF. In our case, the patient was relatively young, so we believed it would be worth trying to retrieve the IVCF implanted 13 years earlier, even though it could carry some risk of vessel rupture or injury.^14)^ Generally speaking, if we succeed in removing the IVCF, patients could be free from the need for anticoagulation, which seems to be a huge advantage to those patients. However, this patient had May–Thuner syndrome on his left CIV. Therefore, he may not be the suitable candidate to gain the full advantage in terms of discontinuing anticoagulation. Anzai et al. reported the beneficial impact of an advanced technique for IVCF retrieval, which improved the retrieval success rate from 75% to 98% in 107 consecutive patients.^15)^ Therefore, we tried to retrieve this aged IVCF using an advanced technique.

## Conclusions

We successfully treated the patient who developed IVCF thrombotic occlusion 13 years after the IVCF implantation. Even under continuous anticoagulation, IVCF thrombotic occlusion might occur in the remote period. In those cases, we need to clear the thrombus using various treatment options, such as CDT or aspiration. Furthermore, in selected patients with IVCF-associated thrombotic occlusion, IVCF retrieval may be considered even after a long indwelling period, provided that the risks and benefits are carefully assessed and advanced retrieval techniques are available.
